# Mitochondria-mediated apoptosis in mammals

**DOI:** 10.1007/s13238-014-0089-1

**Published:** 2014-07-31

**Authors:** Shunbin Xiong, Tianyang Mu, Guowen Wang, Xuejun Jiang

**Affiliations:** 1 Department of Genetics, The University of Texas, M.D. Anderson Cancer Center, Houston, TX 77030 USA; 2 Institute of Cancer Stem Cell, Dalian Medical University Cancer Center, 9 Lvshun Road South, Dalian, 116044 China; 3 Department of Bone and Soft Tissue Tumors, Tianjin Medical University Cancer Institute & Hospital, Tianjin, 300060 China; 4 Cell Biology Program, Memorial Sloan-Kettering Cancer Center, New York, NY 10065 USA

**Keywords:** apoptosome, Bcl-2 family, IAPs, IAP antagonists, cancer therapy

## Abstract

The mitochondria-mediated caspase activation pathway is a major apoptotic pathway characterized by mitochondrial outer membrane permeabilization (MOMP) and subsequent release of cytochrome *c* into the cytoplasm to activate caspases. MOMP is regulated by the Bcl-2 family of proteins. This pathway plays important roles not only in normal development, maintenance of tissue homeostasis and the regulation of immune system, but also in human diseases such as immune disorders, neurodegeneration and cancer. In the past decades the molecular basis of this pathway and the regulatory mechanism have been comprehensively studied, yet a great deal of new evidence indicates that cytochrome *c* release from mitochondria does not always lead to irreversible cell death, and that caspase activation can also have non-death functions. Thus, many unsolved questions and new challenges are still remaining. Furthermore, the dysfunction of this pathway involved in cancer development is obvious, and targeting the pathway as a therapeutic strategy has been extensively explored, but the efficacy of the targeted therapies is still under development. In this review we will discuss the mitochondria-mediated apoptosis pathway and its physiological roles and therapeutic implications.

## INTRODUCTION

The term “apoptosis” was originally coined to describe a specific type of cell death characterized by specific cellular morphological changes, including membrane blebbing, cell shrinkage, nuclear fragmentation, chromatin condensation, and chromosomal DNA fragmentation. (Kerr, 2002; Kerr et al., 1972; Taylor et al., 2008). The nature of apoptosis as a process of “programmed” cell death was established at molecular level mainly by two waves of studies; the discovery of the oncogene product Bcl-2 as an inhibitor of apoptosis, by Korsmeyer, Cory, and others (Bakhshi et al., 1985; Hockenbery et al., 1990; Vaux et al., 1988), and the *C. elegans* genetic studies by Horvitz and colleagues leading to the identification of a pathway controlling development-associated death of a group of cells in the organism (Ellis and Horvitz, 1986; Horvitz, 1999; Horvitz et al., 1994). The prominent role of mitochondria in apoptosis was subsequently unveiled by Xiaodong Wang and colleagues through their discovery of the cytochrome *c*-mediated caspase activation pathway (Li et al., 1997; Liu et al., 1996; Zou et al., 1997).

## THE BCL-2 FAMILY PROTEINS IN MITOCHONDRIAL APOPTOSIS

**Figure 1:**
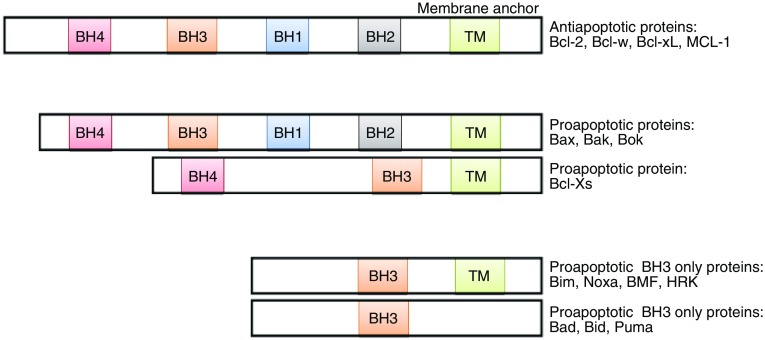
**Schemes showing three groups of Bcl2- family proteins**. BH: Bcl-homology domain; TM: transmembrane domains

**Figure 2:**
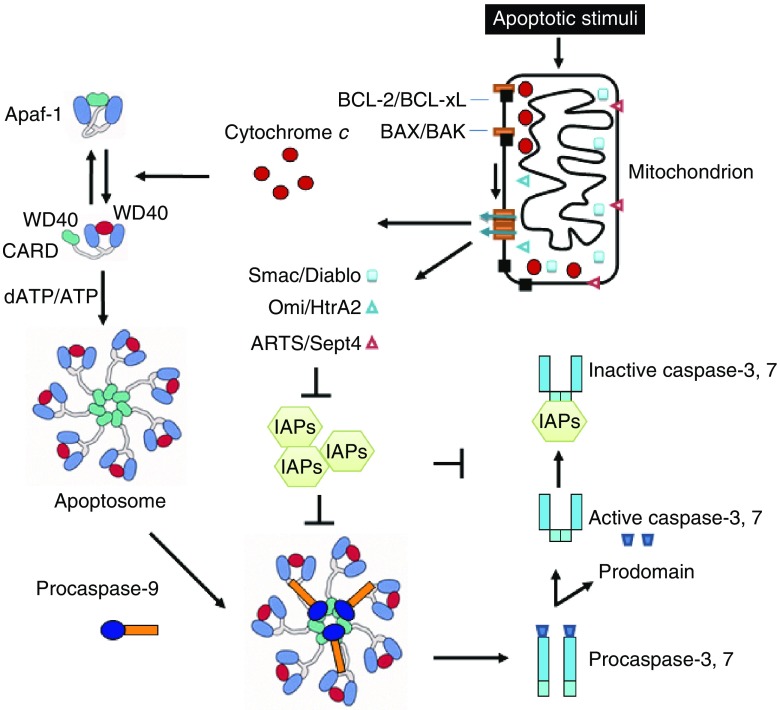
**An overview of the mitochondria-mediated caspase activation pathway**. Upon apoptotic stimuli such as DNA damage, growth factor deprivation, etc. BAX/BAK form oligomeric complexes to mediate cytochrome *c* release from the mitochondria to the cytosol. The released cytochrome *c* forms the apoptosome with Apaf-1 and subsequently activates the initiator caspase, caspase-9, which cleaves and activates effector caspases, caspase-3 and caspase-7, leading to ultimate apoptotic cell death. Other proapoptotic proteins including Smac, Omi, and ARTS also function to repress IAPs to enhance apoptosis. WD40: WD40 repeat domain; CARD: a caspase recruitment domain

The first regulatory step for mitochondrial apoptosis is mediated by Bcl-2 family proteins. Bcl-2, also known as B-cell lymphoma 2, was the first member identified as an apoptosis inhibitory protein overexpressed in human follicular B-cell lymphomas due to t(14;18) chromosomal translocation (Bakhshi et al., 1985; Tsujimoto et al., 1985). Subsequently, three major mammalian groups of Bcl-2 family proteins have been identified. The original pro-survival group includes Bcl2, Bcl-xL, Mcl-1, etc.; an opposite functional group also called pro-apoptotic BH123 protein group includes Bax and Bak; and the third group called apoptosis initiator group is made of BH3 domain-only proteins including Bad, Bid, Bim, Puma, and Noxa (Fig. 1). Without apoptotic stress, Bcl-2 and Bcl-xL (pro-survival) form heterodimers with Bax and Bak (pro-apoptotic) to maintain the outer mitochondrial membrane (OMM) integrity and block mitochondrial apoptosis. In the presence of apoptotic stimuli, the expression of pro-apoptotic proteins Bax and/or BH3-only proteins (apoptosis initiator) increased, following which they bind to pro-survival Bcl-2 proteins to release Bax/Bak from inhibition. Free Bax and Bak form oligomers, leading to cytochrome *c* release from the intermembrane space of mitochondria to the cytoplasm, likely by forming a channel in OMM. The released cytochrome *c* activates the caspase cascade to induce apoptosis (Hardwick and Soane, 2013) (Fig. 2).

To understand the roles of Bcl-2 family protein *in vivo*, many mouse models have been developed. Loss of *Bcl-2* in mouse results in numerous defects, including growth retardation, short life span, polycystic kidney, apoptosis-induced atrophy in thymus and spleen (Kamada et al., 1995). Bcl-2 null mice also show defects in subpopulation of neurons during neonatal period (Michaelidis et al., 1996). Additionally, mice lacking *Bcl-xL* show early embryonic lethality due to the excess apoptosis of immature neurons in brain, spinal cord, and erythroid cells in the liver, indicating the role of *Bcl-xL* during neuron and erythrocyte maturation (Motoyama et al., 1999; Motoyama et al., 1995). The data strongly support the inhibitory roles of *Bcl-2* and *Bcl-xL* in apoptosis, though the function may be tissue and developmental stage specific. On the contrary, the Bax/Bak knockout mice fail to promote MOMP and are resistant to various apoptotic stimuli, demonstrating the essential role of BAK and BAX in mitochondria-mediated apoptosis (Lindsten et al., 2000; Wei et al., 2001). Deletion of any single BH3-only gene in mice, on the other hand, does not result in obvious developmental defects (Ren et al., 2010; Villunger et al., 2011), although *Bid* deletion inhibits Fas-induced apoptosis in certain cell types (Yin et al., 1999). Intriguingly, mice with *Bid*, *Bim*, and *Puma* triple knockout showed embryonic lethality, and a subset of the viable triple null mice displayed similar developmental defects to those of *Bax-*/*-Bak-*/*-* mice with persistent interdigital webs of skin on their feet and imperforate vaginas, indicating these three BH3-only proteins in combination are essential for Bak/Bax activation (Ren et al., 2010; Villunger et al., 2011).

## THE APOPTOSOME FORMATION AND CASPASE CASCADE AFTER CYTOCHROME *C* RELEASE

The second regulatory step of mitochondrial apoptosis is the formation of apoptosome. After MOMP is triggered, mitochondrial proteins such as cytochrome *c* can be released to the cytoplasm. The released cytochrome *c* binds to apoptotic protease activating factor-1 (Apaf-1), and activates nucleotide exchange activity of Apaf-1. The ADP/dADP-associated, inactive Apaf-1 becomes active, ATP/dATP-bound Apaf-1, and forms the apoptosome, a wheel-shaped homo-heptameric Apaf-1 complex. Interestingly, although the hydrolysis of dATP by Apaf-1 was initially thought to be essential for apoptosome function (Zou et al., 1997; Zou et al., 1999), more precise analysis demonstrate that dATP-binding but not hydrolysis is required for apoptosome function (Jiang and Wang, 2000). C-terminal WD40 repeats of Apaf-1 have auto-inhibitory activity, and either cytochrome *c* binding or deletion of these repeats can activate Apaf-1 (Hu et al., 1998; Riedl et al., 2005). Also it is important to have exogenous dATP/ATP present when cytochrome *c* binds to Apaf-1 to avoid the formation of non-functional aggregates (Kim et al., 2005). When activated Apaf-1 forms apoptosome, it binds and cleaves initiator procaspase-9, and converts it to an active form (Fig. 2).

Although the proteolytic processing of a caspase is usually necessary and sufficient for its activation (Thornberry and Lazebnik, 1998), cleaved caspase-9 needs to be associated with the apoptosome complex to be active (Jiang and Wang, 2000; Rodriguez and Lazebnik, 1999). In addition, even when all the possible cleavage sites of caspase-9 are mutated, the uncleaved caspase-9 can still be activated if it is associated with the functional apoptosome (Acehan et al., 2002; Jiang and Wang, 2000), indicating that proteolytic cleavage of caspase-9 is not required for its activation. Therefore, the holoenzyme formed by the apoptosome complex and caspase-9 is critical to activate downstream effector caspases, such as caspase-3, and caspase-7. On the other hand, although caspase-9 cleavage is not required for its activity, the cleavage significantly enhances the enzymatic activity of apoptosome-associated caspase-9 (Zou et al., 2003). Further, caspase-9 can undergo an autocatalysis process which does not change its own enzymatic activity, but is important for its regulation by inhibitors of apoptosis proteins (IAPs) (Twiddy and Cain, 2007), as we will discuss later.

The importance of these key components in mitochondrial apoptotic pathway has been validated by mouse model studies. Cytochrome *c* with a K72A mutation is defective in interaction with Apaf-1, but retains its respiration-associated function (Yu et al., 2001). A knock-in mouse with cytochrome *c* K72A mutation shows strong resistance to DNA damage-induced apoptosis (Hao et al., 2005). *Apaf-1* or *caspase-9* knockout mice have the similar developmental defects as *caspase-3* null mice with central nervous system and lymphocyte homeostasis defects caused by apoptotic deficiency (Cecconi et al., 1998; Hakem et al., 1998; Kuida et al., 1998; Kuida et al., 1996; Woo et al., 1998; Yoshida et al., 1998). Thus, the essential roles of cytochrome *c*, Apaf-1, caspases in this apoptotic pathway have been confirmed *in vivo*.

## THE INHIBITORS OF APOPTOSIS (IAPS)

**Figure 3:**
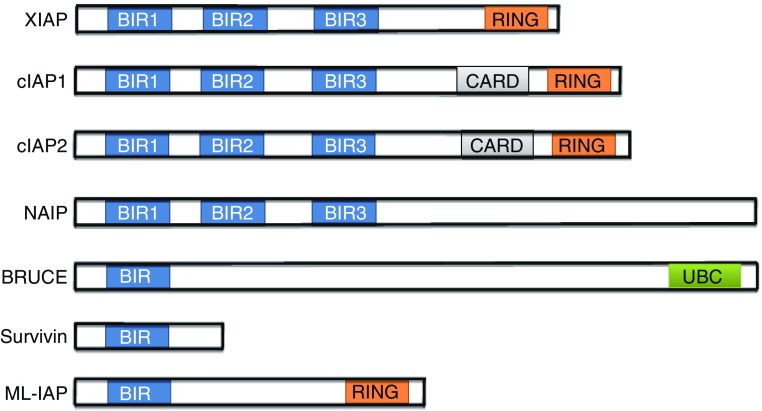
**The structure of IAP family proteins**. The IAP family protein has at least one baculovirus inhibitor of apoptosis protein repeat (BIR) domain. Several IAPs also contain a RING-zinc finger domain at the carboxy terminus with autoubiquitination and degradation activity. c-IAP1 and c-IAP2 have a caspase recruitment domain (CARD) between the BIR domains and the RING domain. BRUCE contains an ubiquitin-conjugation domain (UBC)

Whereas cytochrome *c* release from mitochondria leads to caspase activation and triggers apoptosis, the process is also tightly controlled by other endogenous regulators. The inhibitors of apoptosis (IAPs) family of proteins have BIR (baculovirus IAP repeats) domains. The BIR domain was originally discovered in baculovirus proteins (Crook et al., 1993) that can bind to caspases to inhibit their activity (Deveraux et al., 1997; Roy et al., 1997; LaCasse et al., 1998). IAP family proteins in mammals include X-chromosome linked IAP (XIAP), cellular IAP1 and 2 (cIAP1 and cIAP2), neuronal apoptosis inhibitory protein (NAIP), BRUCE (also called Apollon), Survivin, and ML-IAP (Deveraux and Reed, 1999; Dubrez et al., 2013; Harlin et al., 2001; LaCasse et al., 1998; Vucic et al., 2000). Similar to insect IAPs, mammalian IAPs can bind to caspase-3, 7, and 9 to inhibit apoptosis (Chai et al., 2001; Huang et al., 2001) (Fig. 3). Intriguingly, different IAP proteins may interact with a variety of pro-apoptotic proteins in tissue specific manner to inhibit apoptosis induced by diverse signals.

The relevance of IAP family proteins *in vivo* has been demonstrated by many mouse model studies. Survivin is essential in suppressing apoptosis during mouse development, *Survivin* null mice are lethal at early embryonic stage (Uren et al., 2000). Tissue specific deletion of *Survivin* in thymocytes causes mitotic defects and cell death (Okada et al., 2004), clearly indicating that the pro-survival role of Survivin *in vivo*. Similarly, Bruce/Apollon deletion in mouse causes activation of caspases and apoptosis in the placenta and yolk sac, leading to embryonic lethality. *Bruce*/*Apollon*-deficient MEFs are also sensitive to apoptosis (Hao et al., 2004; Ren et al., 2005). However, some IAP family proteins show functional redundancy with other IAP family members *in vivo*. Mice with *XIAP* deletion are normal and have no detectable defect in apoptosis with a compensating up-regulation of *c-IAP1* and *c-IAP2* (Harlin et al., 2001), while mice with deletion of *cIAP1* in combination with *cIAP2* or *XIAP* show embryonic lethality due to cardiovascular defects (Moulin et al., 2012). Although these *in vivo* studies have demonstrated important roles of IAP proteins in development, whether they exert these functions by directly inhibiting caspase activity, particularly, mitochondria-mediated caspase activation, is not defined.

## IAP ANTAGONISTS AND THE INTERACTION WITH IAPS

Intriguingly, there is another family of proteins that functions to antagonize the anti-apoptotic activity of IAP proteins. This group of proteins was originally discovered in Drosophila by genetic screens. Pro-apoptotic genes Reaper, Hid, and Grim (RHG genes) were identified as suppressors of Drosophila IAP1 (dIAP1) (Chen et al., 1996; Goyal et al., 2000; Grether et al., 1995; Vucic et al., 1997; Vucic et al., 1998; Wang et al., 1999; White et al., 1996). The RHG proteins can compete with caspases to interact with the BIR domain of dIAP1 (Goyal et al., 2000). There are no obvious RHG homologous sequences in mammals. The mammalian RHG counterpart proteins were independently purified based on the apoptotic activity from two groups. Smac (second mitochondrial activator of caspases) was identified as a mitochondria-derived caspase activator in addition to cytochrome *c* (Du et al., 2000), and DIABLO was found by XIAP affinity purification (Verhagen et al., 2000). Interestingly, Smac and DIABLO turned out to be the same mitochondrial protein. The N-terminal AVPI motif of Smac/DIABLO specifically interacts with a groove region of the BIR3 domain of XIAP (Liu et al., 2000; Wu et al., 2000), which is sufficient to antagonize the inhibitory activity of BIR3 domain towards caspase-9 (Chai et al., 2000). Subsequently, other IAP antagonists were also identified from mitochondria in mammalian cells. For example, Omi/HtrA2 binds to XIAP, thereby antagonizing caspase-XIAP interaction. Interestingly, Omi/HtrA2 also degrades IAPs through its serine protease activity (Hegde et al., 2002; Martins et al., 2002; Suzuki et al., 2001; van Loo et al., 2002; Verhagen et al., 2002; Yang et al., 2003). ARTS/Sept4 is a septin-like IAP antagonist,which has a unique mechanism to regulate IAPs (Gottfried et al., 2004; Larisch et al., 2000). Unlike Smac and Omi localizing in mitochondria, ARTS is localized on the surface of the mitochondrial outer membrane, allowing it to interact with IAPs independent of MOMP (Edison et al., 2012).

Smac also suppresses the inhibitory activity of XIAP toward caspase-3 by cooperatively interacting with the BIR3 and BIR2 domains of XIAP. Thus, although the multiple BIR-domains of XIAP confer its concurrent inhibitory function to caspase-9 and caspase-3, it also makes the protein highly susceptible to inhibition by Smac (Gao et al., 2007). In addition to the BIR domains, most IAP proteins also have a RING domain with E3 ubiquitin ligase activity, which can cause ubiquitin-mediated degradation of active caspases and SMAC/Diablo (MacFarlane et al., 2002), indicating the RING domain of IAPs is also important for their anti-apoptotic function. Conversely, the serine protease activity of Omi/HtrA2 can also inactivate cIAPs and XIAP by proteolytic cleavage (Yang et al., 2003). Smac/Diablo can promote auto-ubiquination and degradation of cIAPs (Yang and Du, 2004). Thus IAPs and their antagonists have multiple ways *in vivo* to tightly regulate the mitochondrial apoptosis pathway (Figs. 2 and 3).

The function of these IAP antagonists may be redundant or tissue-specific *in vivo* as indicated by mouse models. *Smac*-deficient mice were viable and normal. Cultured *Smac*-null cells show normal response to all apoptotic signals, suggesting other IAP antagonist molecules can compensate the loss of *Smac* (Okada et al., 2002). HtrA2/Omi may work in a tissue specific manner or possess apoptosis-independent functions, since mice lacking *HtrA2/Omi* only show a neurodegenerative disorder similar to a Parkinson phenotype due to the loss of neurons in the striatum (Martins et al., 2004). *Arts*/*Sept4*-null mice show increased numbers of hematopoietic stem and progenitor cells, elevated XIAP protein, increased resistance to cell death, and accelerated tumor development in an Eμ-Myc background. These phenotypes are partially rescued by the inactivation of XIAP (Garcia-Fernandez et al., 2010). Thus, the apoptotic role of ARTS/Sept4 is specific to certain cell lineages and involved in cancer development.

## CROSSTALK OF THE MITOCHONDRIAL PATHWAY WITH THE DEATH RECEPTOR-MEDIATED APOPTOSIS AND NECROSIS

In addition to the mitochondrial pathway, mammalian cells possess the death receptor-mediated apoptotic pathway that is triggered by the tumor necrosis factors (TNF family). The TNF family factors include Fas ligand, TNF-alpha, Apo3L, Apo2L, and TRAIL (TNF-related apoptosis inducing ligand) that can activate their corresponding receptors FasR, TNFR1, DR3, and DR4/DR5 (Ashkenazi et al., 2008; Tait and Green, 2010a). Upon receptor activation, the adaptor molecules such as FAS-associated death domain protein (FADD) are recruited to associate and activate caspase-8 or caspase-10, which leads to the cleavage and activation of caspase-3 and caspase-7. There is crosstalk between mitochondrial and death receptor pathways. Caspase-8/10 can activate mitochondrial apoptosis initiator protein BID, thus forming an amplification loop to enhance the mitochondrial pathway (Li et al., 1998; Luo et al., 1998). Conversely, Bcl-2 overexpression can completely block apoptosis induced by TNF ligands in various cell types known as Type-II cells (Jiang and Wang, 2004; Scaffidi et al., 1998), suggesting the mitochondrial amplification loop is required for sufficient activation of effector caspases by the death receptor pathway. This is further supported by the observation that Smac and Omi are released to antagonize IAPs by caspase-8-activated BID (Jost et al., 2009; Sun et al., 2002). Additionally, Smac/DIABLO overexpression can sensitize cells to TRAIL and overcome TRAIL resistance in malignant glioma xenografts model (Fulda et al., 2002). Small molecules mimicking Smac can sensitize various cell types to both TRAIL- and TNFα-induced apoptosis (Li et al., 2004). Further, in some Type-II cells, cellular apoptosis susceptibility protein (CAS) can be upregulated by death receptor ligands to stimulate of Apaf-1 (Kim et al., 2008). Therefore, the death receptor pathway can enhance the mitochondria-mediated pathway through multiple mechanisms.

While the functional interplay between the mitochondrial pathway and death receptor-mediated apoptosis is well established, recent evidence suggests that the mitochondrial pathway also communicates with death receptor-induced programmed necrosis (also called necroptosis). The typical morphologies of necrosis are the formation of intracellular vacuoles, organelle swelling, and plasma membrane rupture (Chan, 2012). Although necrosis is originally thought to be passive, it has been unambiguously demonstrated that at least at certain contexts, necrosis can be programmed. For example, death receptor-mediated necrosis requires a kinase cascade, including receptor interacting protein (RIP) kinases RIP1 and RIP3, and the effector protein MLKL (Cho et al., 2009; He et al., 2009; Sun et al., 2012; Zhang et al., 2009). Death receptor-mediated necrosis plays an important role during development and maintenance of adaptive immune response (Han et al., 2011; Li et al., 2012; Linkermann and Green, 2014), and there is intimate crosstalk between this pathway and death receptor-mediated apoptosis, an alternative outcome of the death receptor signaling. For example, caspase-8 activity inhibits RIP3-dependent necrosis (Oberst et al., 2011) and RIP3 in turn suppresses death receptor-mediated apoptosis (Newton et al., 2014). Intriguingly, the mitochondrial apoptotic pathway also shares some regulatory components with necrosis. For example, necrosis caused by hepatic and cerebral ischemia/reperfusion is reduced by inhibition of Bax, and the effect is even stronger than that caused by inhibition of initial apoptotic signal, suggesting Bax plays an important role to promote necrotic cell death under this context (Ben-Ari et al., 2007; Hetz et al., 2005). In addition, Bmf, a pro-apoptotic Bcl-2 protein, is another example of mitochondrial pathway regulator that has been implicated in TNFα-induced necrosis (Hitomi et al., 2008).

## CELL FATE DETERMINATION AND NON-CANONICAL FUNCTIONS OF THE MITOCHONDRIAL PATHWAY

It was originally believed that once MOMP is triggered, cells are doomed to die even when downstream caspase activation is completely inhibited (Cheng et al., 2001; Goldstein et al., 2005; Goldstein et al., 2000). However, new evidence shows that cells can survive with partial MOMP and induction of modest cytochrome *c* release. As mentioned previously, cells have developed multiple mechanisms to regulate caspase activation downstream of cytochrome *c* release, which strongly suggests that apoptosis can still be avoided even after cytochrome *c* release. For example, Apaf-1 or caspase-9 knockout mice show the resistance to cell death in the developing neuronal cells (Cecconi et al., 1998; Hakem et al., 1998; Kuida et al., 1998; Yoshida et al., 1998). As both caspase-9 and Apaf-1 function downstream of cytochrome *c* release, these studies demonstrate that deficiencies downstream cytochrome *c* release can also block cell death, and thus cytochrome *c* release is not always the “point of no-return” of mitochondrial apoptosis. Also, when cleaved BID induces modest cytochrome *c* release, if downstream caspase activation is inhibited, the same cells can fully recover and proliferate (Tait et al. 2010b).

Additionally, cytochrome *c* release may have non-apoptotic functions. For example, cytochrome *c*-mediated caspase activation in hippocampal neurons does not lead to apoptosis, yet it is required for brain development and function (Li et al., 2010), indicating that cytochrome *c* release has non canonical functions at least in neurons. Furthermore, caspase activation is also involved in many biological processes, including sperm and red blood cell differentiation (Kuranaga and Miura, 2007; Lamkanfi et al., 2007; Zermati et al., 2001), and axonal pruning (Nikolaev et al., 2009). Interestingly, caspase-3 deficient mice have increased B cells with enhanced proliferation and hyperproliferation under mitogen treatment (Woo et al., 2003), indicating that caspase-3 can also be involved in cell cycle arrest. In these caspase-dependent events, caspase activity does not result in cell death, but is involved in cellular component clearance and loss of cell mass. The mechanisms underlying how cells determine if cytochrome *c*-mediated caspase activation should lead to apoptotic cell death or a specific non-death biological function remain unclear.

Besides non-canonical function of caspases, other members of the mitochondrial pathway are also involved in non-death processes in cells. Some of the Bcl-2 family proteins regulate calcium homeostasis, glucose metabolism, and mitochondrial dynamics (Chen et al., 2004; Danial et al., 2010; Danial et al., 2003; Popgeorgiev et al., 2011; Rolland and Conradt, 2010). Apaf-1 is involved in DNA damage induced cell cycle arrest independent of caspase activation (Zermati et al., 2007). The members of the IAP family, Survivin, is involved in kinetochore function (Skoufias et al., 2000; Speliotes et al., 2000), while cIAP1 and cIAP2 are critical regulators of the NF-κB signaling (Beug et al., 2012). Human NAIP regulates the host response to *L. pneumophila* infection and inhibits apoptosis or promotes pyroptosis in response to specific cellular signals (Katagiri et al., 2012). As proteins of mitochondrial pathway are important for many developmental and cellular events independent of cell death, it is important to determine whether the phenotypes caused by alteration of these proteins are related to mitochondria-mediated apoptosis or their non-canonical functions.

## THE ROLE OF MITOCHONDRIAL APOPTOSIS PATHWAY IN CANCER AND CANCER TREATMENT

As we discussed in previous sections, apoptosis is essential for multiple physiological processes. Because aberrant apoptotic cell death is one of the hallmarks of tumorigenesis and tumor progression, cancer cells develop various mechanisms to deregulate the mitochondrial pathway, which leads to apoptotic resistance and survival advantage.

**Figure 4:**
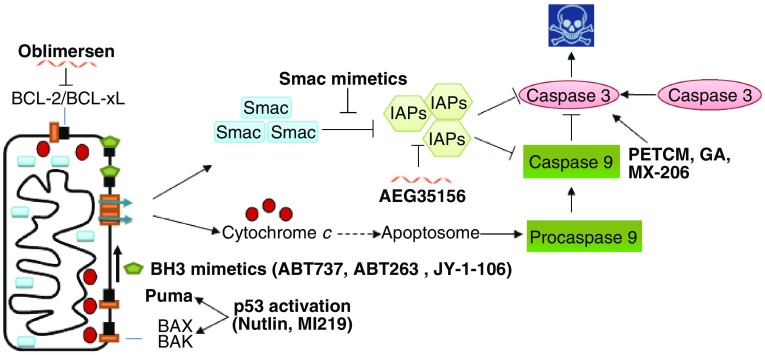
The therapeutic agents developed to target the mitochondrial apoptotic pathway. Oblimersen sodium is a Bcl-2 antisense oligonucleotide compound. BH3 mimetic compounds include ABT- 737, ABT-263, and JY-1-106. Nutlin and MI-219 block Mdm2 and p53 interaction to activate p53 transcription activity to induce the expression of Puma and Bax. Smac mimetics and the antisense oligonucleotide AEG35156 are inhibitors of XIAP. 4-Pyridineethanol (PETCM), gambonic acid, and the gambonic acid derivative MX-206 can activate caspases-3

Many components of the mitochondrial apoptosis pathway are deregulated in cancer cells. The elevated expression of pro-survival Bcl-2 gene has been identified in many different cancers, including melanoma, breast, prostate, chronic lymphocytic leukemia, and lung cancer. The high expression of Bcl-2 imparts therapeutic resistance of these cancer cells. Tremendous effort has been spent on developing drugs to target the Bcl-2 pro-survival family members. The first clinical trial agent that targets Bcl-2 is oblimersen sodium (a Bcl-2 antisense oligonucleotide compound). This oligonucleotide specifically binds to human *bcl-2* mRNA, resulting in its degradation (Herbst and Frankel, 2004) (Fig. 4).

Another strategy to target the Bcl-2 family proteins (Bcl-2, Bcl-w, Bcl-xL, MCL-1) is to develop potent BH3 mimetic compounds. These BH3 mimetic compounds bind the hydrophobic groove of anti-apoptotic Bcl-2 proteins in place of BH3-only proteins, allowing Bax and other pro-apoptotic proteins to induce MOMP and apoptotic death. ABT-737 and the orally form ABT-263 developed by Abbott are successful examples. ABT-263 induces tumor regression in the xenograft models of small cell lung cancer and acute lymphoblastic leukemia (Ackler et al., 2008; Tse et al., 2008). More recently, another BH3 mimetic compound JY-1-106 is demonstrated to induce apoptosis in lung cancer, colon cancer, and mesothelioma (Cao et al., 2013).

On the other hand, the pro-apoptotic Bax and BH3-only proteins Puma, Noxa are the transcriptional targets of p53 tumor suppressor. Since it is well known that one of the mechanisms for p53 to suppress tumorigenesis is mediated by its apoptosis function, activation of p53 pathway can be an appealing therapeutic strategy to treat cancer. The most common mechanism to inactivate p53 function in human tumors is missense mutations; several compounds have been developed to restore activity of mutant p53. A synthetic 22-mer peptide corresponding to the carboxy-terminal amino acid residues 361–382 of p53 was the first compound identified to restore mutant p53 activity in tumor cells thereby inducing apoptosis (Selivanova et al., 1997). PRIMA-1 has been shown to have a similar function (Bykov et al., 2002). More recently, a compound (NSC319726) from the thiosemicarbazone family was shown to specifically restore the activity of p53^R175H^ mutation (Yu et al., 2012). However, all these compounds still need to be tested in patients for efficacy. Additionally, other mechanisms, such as overexpression of p53 negative regulators Mdm2 and Mdm4, have been proven to be alternative ways to inactivate wild type p53 function in human tumors (Oliner et al., 1992; Toledo and Wahl, 2007; Wade et al., 2010). In tumors with wild type p53, activation of p53 to induce apoptosis can be achieved by blocking Mdm2 or Mdm4 binding to p53 (Martins et al., 2006; Shchors et al., 2013; Ventura et al., 2007; Wang et al., 2011; Xue et al., 2007). Several chemicals, such as Nutlin and MI-219, have been developed to block the interaction between Mdm2 and p53 (Shangary et al., 2008; Vassilev et al., 2004). Chemicals targeting Mdm4 are still under development.

Increased expression of pro-apoptotic proteins, such as Apaf-1 and Smac are associated with longer survival in cancer patients (Endo et al., 2009; Huang et al., 2010; McIlwain et al., 2013; Provencio et al., 2010; Strater et al., 2010; Zlobec et al., 2007). Conversely, over-expression of IAP proteins are frequently detected in various human cancers and associated with poor prognosis (Barrett et al., 2011; Fulda and Vucic, 2012; Mizutani et al., 2007; Tamm et al., 2000). Thus, blocking IAP proteins in human tumors may improve patient survival. Smac mimetics induce apoptosis through their ability to suppress IAPs by direct inhibition and/or proteasomal degradation of some members of the IAP family. These compounds can target cancer cells with IAPs overexpression, and some of these compounds are currently in clinical trials (Chen and Huerta, 2009; Fulda and Vucic, 2012; Lu et al., 2008). Also an antisense oligonucleotide against XIAP (AEG35156) has been developed to treat patients with pancreatic, breast, non-small cell lung cancer, AML, and lymphoma (Mahadevan et al., 2013; Schimmer et al., 2009).

Additionally, decreased expression of caspase-3 is frequently observed in cancer cells and is associated with chemoresistance. Conversely, activation of caspase-3 often increases cancer cell sensitivity to apoptosis (Devarajan et al., 2002; Guicciardi and Gores, 2013). 4-pyridineethanol (PETCM), gambonic acid, and the gambonic acid derivative MX-206 were identified by high-throughput screens for caspases 3 activation *in vitro*. Some of these molecules have been reported to induce apoptosis in cancer cell lines (Jiang et al. 2003; Zhang et al. 2004; Fischer and Schulze-Osthoff 2005).

More recently, many studies showed that combination therapies can achieve better therapeutic effect. When ABT-737 is administrated together with paclitaxel, it can enhance the cytotoxic effect of paclitaxel (Lieber et al., 2011). Although the alkylating agent temozolomide (TMZ) is commonly used in treating melanoma, it has low response rate by itself. Combining ABT-737 with TMZ can induce strong apoptosis in multiple human melanoma cell lines and in a mouse xenograft model at much lower concentrations (Reuland et al., 2011). To activate apoptosis in tumors, SMAC mimetic compounds (SMCs) have disappointing effects as single agents in tumors with low expression of death-inducing proteins. However, Smac mimetic BV6, which antagonizes XIAP, cIAP1, and cIAP2, together with the demethylating agent 5-azacytidine or 5-aza-2’-deoxycytidine can induce cell death more efficiently in otherwise resistant AML cells (Steinhart et al., 2013). In conclusion, many drugs are under development to target different components of the mitochondrial apoptotic pathway to treat cancer patients (Fig. 4). Further investigation is needed to improve the efficacy of these leading compounds in humans.

## PERSPECTIVE

Tremendous progresses have been made for our understanding of the molecular mechanisms and biological function of mitochondrial apoptotic pathway, leading to potential therapeutic development to target the components of the pathway. Recent work also led to the discovery of novel functional interactions between the mitochondrial pathway and other death pathways, including programmed necrosis. In addition, it becomes clear that the function of the mitochondrial pathway is context-dependent and cell death is not necessarily always its “intended” biological outcome. Therefore, it is important to decode the context-specific regulatory mechanisms of the pathway, and to dissect the function of the pathway in a spatial and temporal specific manner *in vivo.* Further investigation is needed in order to achieve a more complete understanding of the mechanisms and biology of the mitochondria-mediated caspase activation pathway, and for eventual therapeutic application targeting this important pathway.

## ABBREVIATIONS

Apaf-1: apoptotic protease activating factor-1
BIR: baculovirus IAP repeats
CAS: cellular apoptosis susceptibility protein
FADD: FAS-associated death domain protein
IAPs: inhibitors of apoptosis proteins
MOMP: mitochondrial outer membrane permeabilization
NAIP: neuronal apoptosis inhibitory protein
OMM: outer mitochondrial membrane
RIP: receptor interacting protein
XIAP: X-chromosome linked IAP
